# DPEP Inhibits Cancer Cell Glucose Uptake, Glycolysis and Survival by Upregulating Tumor Suppressor TXNIP

**DOI:** 10.3390/cells13121025

**Published:** 2024-06-12

**Authors:** Qing Zhou, Trang Thi Thu Nguyen, Jeong-Yeon Mun, Markus D. Siegelin, Lloyd A. Greene

**Affiliations:** 1 Department of Pathology and Cell Biology, Vagelos College of Physicians and Surgeons, Columbia University Irving Medical Center, New York, NY 10032, USA; qz2266@cumc.columbia.edu (Q.Z.); thithutrang.nguyen@nyulangone.org (T.T.T.N.); jm5576@cumc.columbia.edu (J.-Y.M.); ms4169@cumc.columbia.edu (M.D.S.); 2 Ronald O. Perelman Department of Dermatology, Perlmutter Cancer Center, NYU Grossman School of Medicine, NYU Langone Health, New York, NY 10016, USA

**Keywords:** glucose uptake, glycolysis, TXNIP, CEBPB, CEBPD, ATF5, cell-penetrating, apoptosis, apoptotic death

## Abstract

We have designed cell-penetrating peptides that target the leucine zipper transcription factors ATF5, CEBPB and CEBPD and that promote apoptotic death of a wide range of cancer cell types, but not normal cells, in vitro and in vivo. Though such peptides have the potential for clinical application, their mechanisms of action are not fully understood. Here, we show that one such peptide, Dpep, compromises glucose uptake and glycolysis in a cell context-dependent manner (in about two-thirds of cancer lines assessed). These actions are dependent on induction of tumor suppressor TXNIP (thioredoxin-interacting protein) mRNA and protein. Knockdown studies show that TXNIP significantly contributes to apoptotic death in those cancer cells in which it is induced by Dpep. The metabolic actions of Dpep on glycolysis led us to explore combinations of Dpep with clinically approved drugs metformin and atovaquone that inhibit oxidative phosphorylation and that are in trials for cancer treatment. Dpep showed additive to synergistic activities in all lines tested. In summary, we find that Dpep induces TXNIP in a cell context-dependent manner that in turn suppresses glucose uptake and glycolysis and contributes to apoptotic death of a range of cancer cells.

## 1. Introduction

The leucine zipper transcription factors ATF5 (Activating Transcription Factor 5), CEBPB (CCAAT Enhancer Binding Protein Beta) and CEBPD (CCAAT Enhancer Binding Protein Delta) play significant roles in a wide variety of malignancies by promoting cancer formation, growth, survival, metastasis and treatment resistance [1,2,3,4,5,6,7,8,9]. To target these factors, we designed a series of peptides that contain portions of the leucine zippers of each protein fused to an N-terminal penetratin cell-penetrating domain [1,3,10,11,12]. As such, the peptides associate with the leucine zippers of their obligate dimerization partners, but since they lack DNA-binding domains, they act as dominant-negative decoys to suppress their activities [1,7]. The peptides, designated as CP-dn-ATF5 (cell-penetrating dominant-negative ATF5), Bpep and Dpep, promote apoptotic death of a remarkably wide range of tumor cell types both in culture and in animal models [3,7,10,11,12,13,14]. They also have a high degree of safety, with no apparent effects on non-transformed cells in culture or in rodents [3,10,11,12,13]. CP-dn-ATF5 binds and suppresses activity of CEBPB and CEBPD, while Dpep and Bpep appear to associate with and inhibit ATF5 as well as CEBPB and CEBPD [1,7].

Though the peptides promote apoptotic tumor cell death [11,12,13,15], the upstream mechanisms leading to such death are unclear. In addition, it is not fully understood how the peptides are capable of affecting such a wide range of cancer cell types. To address these issues, we compared the transcriptional profiles of six divergent tumor cell types treated with and without Dpep [16]. The results suggested a mechanism in which the peptide disrupts multiple pathways, some of which are shared and some of which are cell context-dependent, and these converge on activation of apoptosis.

We reasoned that among the relevant pathways affected by Dpep would be ones that are widely shared by various tumor cell types and that distinguish tumor cells from most non-transformed cells. One such candidate is dependence on aerobic glycolysis. Such dependence, often designated as the “Warburg effect”, has been widely studied and has been identified as a potential, though challenging, target for therapeutic treatment of cancers [17,18,19]. Moreover, several studies have documented positive regulation of glycolysis by Dpep targets CEBPB and CEBPD [20,21,22,23,24,25]. Here, we describe context-dependent suppression of tumor cell glycolysis by Dpep, the mechanisms by which this occurs via suppression of glucose uptake by upregulation of the tumor suppressor TXNIP and its relevance to promotion of apoptotic death. Given Dpep’s effects on glycolysis, we also evaluate the complementary combination of Dpep with several drugs that inhibit oxidative phosphorylation.

## 2. Materials and Methods

### 2.1. Cell Culture

T98G, LN229, MDA-MB-231, MCF7, A549, HCT116, A375 and MCF10A cells were purchased from and authenticated by the ATCC. GBM22 cells (a patient-derived xenograft line, WHO grade IV, were obtained from Dr. Jann Sarkaria (Mayo Clinic, Rochester, MN, USA). All lines were examined using a Universal Mycoplasma Detection Kit (ATCC, Manassas, VA, USA; #30-1012K) and were confirmed to be free from mycoplasma contamination. The cells were cultured in DMEM supplemented with 10% FBS and 100 U/mL Penicillin–Streptomycin. For experiments related to cell number, survival or protein/mRNA expression, cells were seeded onto 96- or 6-well tissue culture plates pre-coated overnight with a 0.1 µg/µL poly-D-lysine solution and then air-dried for 15 min, unless stated otherwise.

### 2.2. Peptides and Reagents

Dpep and mutated peptides were purchased as acetate salts from Alan Scientific (Laurel, MD, USA) with the following sequences:

Dpep: RQIKIWFQNRRMKWKKLVELSAENEKLHQRVEQLTRDLAGLRQFFK;
Dpep-mut: RQIKIWFQNRRMKWKKLVEGSAENEKGHQRVEQGTRDGAGRQFFK.

Peptides were dissolved in 10% glycerol in PBS at a pH of 7.2 and stored as 2 mM aliquots at −80°C until dilution for use in experiments.

Metformin hydrochloride (Sigma-Aldrich, St. Louis, MO, USA; #1115-70-4) was prepared as a stock solution at 1M by dissolving the powder in distilled water for subsequent dilution. Atovaquone 10 mM stock solution in DMSO was purchased from Selleckchem (#s3079) and further diluted to the specified concentrations. The final concentration of DMSO in cell culture was maintained below 0.1%.

### 2.3. Cell Viability

Cells were initially seeded into 96-well plates at a density of 1 × 10^4^ cells/well, with each well containing 0.1 mL DMEM supplemented with 10% FBS. After an overnight incubation under consistent culture conditions, the medium in each well was replaced with DMEM containing 2% FBS, along with the specified concentrations of Dpep, metformin or atovaquone, individually or in combination, and maintained for an additional 5 days. Cell viability was determined through cell counting using either a hemocytometer or a Countess II automated cell counter (Life Technologies, Carlsbad, CA, USA). All assays were conducted in triplicate.

### 2.4. Glycolytic Activity

Glycolytic activity was assessed using the Seahorse XF Glycolysis Stress Test Kit (Agilent Technologies, Cedar Creek, TX, USA; #103017-100) following the protocol provided by the supplier. Cells were seeded in triplicate at a density of 15,000 cells per well in an 8-well Seahorse XFp Cell Culture Miniplate (Seahorse XFp FluxPak, Agilent Technologies, #103022-100). The medium was then replaced the next day with DMEM plus 2% FBS and the indicated concentrations of Dpep or Dpep-mut. Analyses were conducted 24 or 48 h later using a Seahorse XFp extracellular Flux Cartridge (Seahorse XFp FluxPak). To assess glycolysis, cells were incubated in Seahorse XF base medium containing 1 mM l-glutamine in a CO_2_-free incubator at 37 °C for 1 h prior to the assay. The extracellular acidification rate (ECAR) was measured at baseline and after exposure to 10 mM glucose, 1 µM oligomycin and 50 mM 2-deoxyglucose. Glycolysis was calculated using ECAR readings based on the manufacturer’s algorithms. Specifically, ECAR after glucose addition defines glycolysis, and ECAR following oligomycin indicates maximum glycolytic capacity. The difference between the glycolytic capacity and the glycolysis rate represents the glycolytic reserve. After each experiment, cell numbers in each well were measured for normalization using a CyQUANT cell proliferation kit (Invitrogen, Carlsbad, CA, USA; #C7026).

### 2.5. Glucose Uptake

Glucose uptake was assessed using the glucose uptake fluorometric assay kit (Sigma-Aldrich, St. Louis, MO, USA; #MAK084). Equal aliquots (200 µL) of cells from the same parent plate were distributed into two separate 96-well plates for distinct purposes: one for assessing glucose uptake and the other for cell number counting. In the glucose uptake plate, cells were seeded in duplicate at a density of 1 × 10^4^ cells per well and subjected to treatment with Dpep at the specified concentrations for 24 h. Following serum deprivation overnight followed by glucose starvation for 40 min in KRPH buffer (20 mM HEPES, 5 mM KH_2_PO_4_, 1 mM MgSO_4_, 1 mM CaCl_2_, 136 mM NaCl, 4.7 mM KCl, pH 7.4) containing 2% BSA, cells were stimulated with or without 1 µM insulin for 20 min and then incubated with 10 µL of 10 mM 2-deoxyglucose for an additional 20 min. Cell lysis was then performed using 80 µL per well of extraction buffer provided in the assay kit followed by 1 cycle of freeze–thawing in a dry ice-ethanol bath and heating to 85 °C for 40 min. After neutralization with 10 µL of buffer provided with the kit and centrifugation at 13,000× *g* for 5 min, the supernatant was mixed with 50 µL of Master Reaction Mix (also provided by the kit) and incubated for 40 min at 37 °C in the dark. Fluorescence intensity was then measured in a Tecan Infinite M200 Multi-Detection Plate Reader at λex = 535/λem = 587 nm. In the cell counting plate, cells were seeded in triplicate at the same density as above and underwent identical treatment as in the glucose uptake plate. Cell numbers at the end of treatment were determined by hemocytometer cell counting. Glucose uptake was quantified by comparing the accumulated 2-DG6P levels in the samples to a standard curve based on its fluorescence intensity and normalized by the corresponding cell counts for each condition.

### 2.6. siRNA Transfections

Transfections were performed with Oligofectamine™ (Invitrogen, #12252-011) according to the manufacturer’s instructions. Following a 48 h transfection period, cells were prepared for utilization in subsequent assays measuring cell viability, apoptosis, glycolysis or glucose uptake. siRNAs were as follows: Silencer™ Select Negative Control No. 2 siRNA (Invitrogen, #439084); Silencer™ Select siRNA TXNIP-1 (Invitrogen, #s20878); Silencer™ Select siRNA TXNIP-2 (Invitrogen, #s20879).

### 2.7. qPCR

Cells were seeded into 6-well plates and lysed after 48 h of transfection as above. Total RNA purification, cDNA synthesis and qPCR were performed following previously established protocols (12) using the following primer pairs, with values normalized to 18S ribosomal RNA:

18S ribosomal RNA Forward primer: 5′-AGTCCCTGCCCTTTGTACACA-3′;
18S ribosomal RNA Reverse primer: 5′-GATCCGAGGGCCTCACTAAAC-3′;
TXNIP Forward primer: 5’-ACAGAAAAGGATTCTGTGAAGGTGAT-3′;
TXNIP Reverse primer: 5’-GCCATTGGCAAGGTAAGTGTG-3′.

### 2.8. Western Immunoblotting

Cells were plated into 6-well plates and subjected to treatment with or without 20 µM Dpep for 48 h. TXNIP protein expression was determined by Western blot analysis as described previously (12). Signals were detected using a CCD camera system (Azure C300 imager, Azure Biosystems, Dublin, CA, USA). Western blot results were quantified using ImageJ (version 1.53m) for band intensity analysis, with normalization to actin as the loading control.

The following antibodies were used: rabbit anti-TXNIP (Cell Signaling Technology, Danvers, MA, USA; #14715), mouse anti-ACTIN (Cell Signaling Technology, #3700).

### 2.9. Plate-Seq Analysis

Plate-seq data were obtained and subjected to bioinformatic analysis as previously described [6]. All raw and processed Plate-seq data associated with this study are available at the Gene Expression Omnibus under accession GSE244579.

## 3. Results

### 3.1. Dpep Promotes Context-Dependent Suppression of Glycolysis in Diverse Cancer Cell Lines

**Figure 1:**
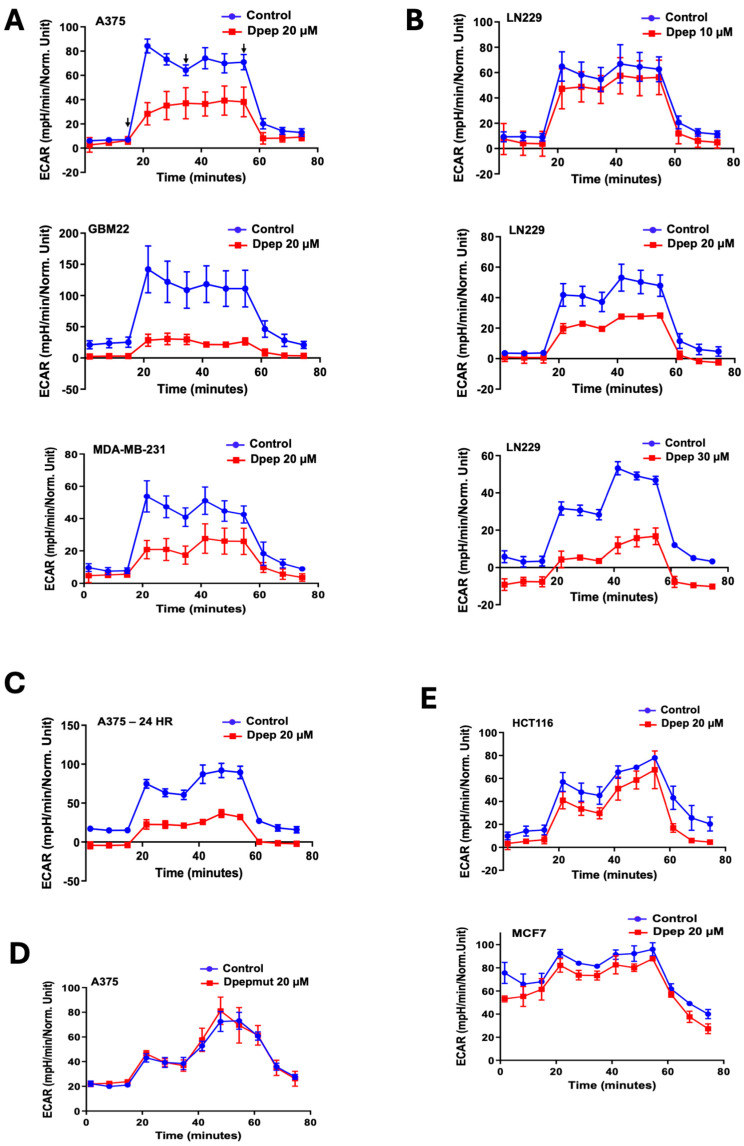
Dpep suppresses glycolysis in a cell context-dependent manner. (**A**) A375, MDA-MB-231 and GBM22 cells were treated with vehicle or 20 µM Dpep for 48 h and then subjected to Seahorse XFp glycolysis stress assays to measure the extracellular acidification rate (ECAR). Assays were carried out in triplicate, with data shown ± SD. Similar results were achieved in 2–3 independent experiments. Arrows in the A375 panel indicate (from the left) the times of addition of glucose, oligomycin and 2-deoxyglucose. Treatment was identical in all other experiments. (**B**) LN229 cells were treated as in A, except that a dose–response study was carried out that included 10, 20 and 30 µM Dpep. (**C**) A375 cells were treated as in A, except that exposure to Dpep was for 24 h. (**D**) A375 cells were treated as in A, but with Dpep-mut, a form of Dpep mutated to block its interaction with target proteins and, therefore, its activity. (**E**) HCT116 and MCF7 cells were treated as in panel A. Note that Dpep did not significantly suppress ECAR in these lines.

To determine whether Dpep affects glycolysis in cancer cells, we used Seahorse XF glycolysis rate assays to measure the extracellular acidification rate (ECAR) of cultures treated with vehicle or 20 µM Dpep for 48 h. The latter concentration shows minimal cell death at 48 h and reduces cell numbers by about 50% by 5–6 days [12]. MDA-MB-231 triple-negative breast cancer cells, GBM22 PDX-derived glioblastoma cells, LN229 glioblastoma cells and A375 melanoma cells all showed significant decreases in glycolysis under these conditions (Figure 1A,B). A dose–response study with LN229 cells showed increasing suppression across a dose range of 10–30 µM Dpep (Figure 1B). Using A375 cells, we also found suppression of glycolysis at 24 h comparable to that seen at 48 h (Figure 1C). In the same line, a mutated control form of the peptide (Dpep-mut) in which key lysines in the zipper domain were replaced with glycine residues to prevent association with targets [12] had no effect on glycolysis after 48 h of treatment (Figure 1D).

We also noted that similar exposure to Dpep did not significantly affect glycolysis in several tumor cell lines, namely, HCT116 colon cancer cells and MCF7 breast cancer cells (Figure 1E). Thus, the capacity of Dpep to suppress glycolysis appears to be relatively widespread, but not universal among cancer cell lines.

### 3.2. Dpep Exhibits Context-Dependent Suppression of Glucose Uptake

**Figure 2:**
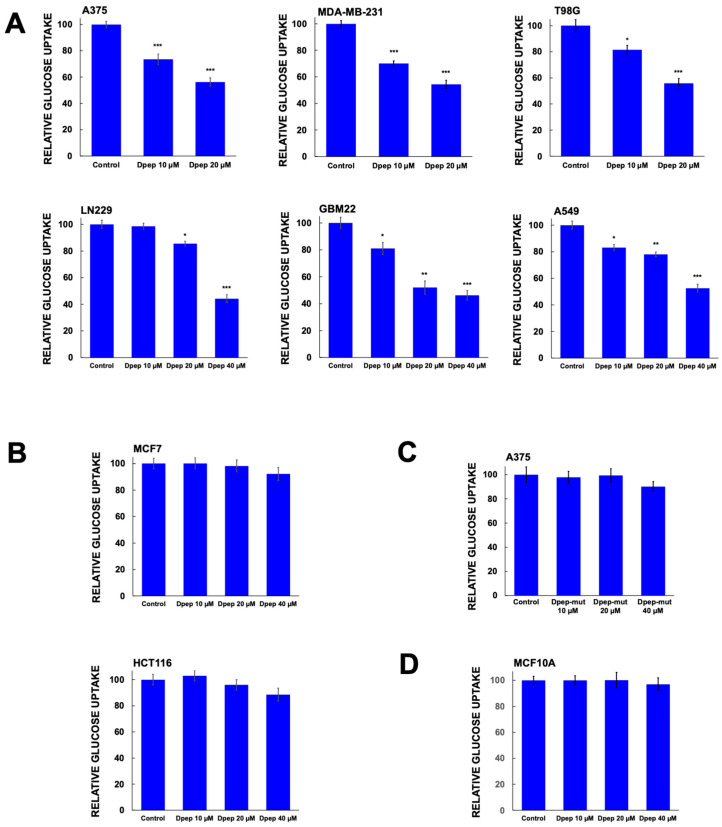
Dpep suppresses glucose uptake in a cell context-dependent manner. (**A**) A375, MDA-MB-231, T98G, A549, LN229 and GBM22 cells were treated with Dpep at the indicated doses of 0–40 µM for 24 h and then assessed for uptake of 2-deoxyglucose. Assays were carried out in duplicate with at least 2 independent experiments. Values are shown as means ± SEM. (**B**) MCF7 and HCT116 were treated with Dpep and assessed for glucose uptake as in panel (**A**). (**C**) A375 cells were treated with Dpep-mut and assessed for glucose uptake as in panel (**A**). (**D**) MCF10A cells were treated with Dpep and assessed for glucose uptake as in panel (**A**). Values represent means ± SEM and represent 2–3 independent experiments, each carried out in duplicate. *** *p* ≤ 0.0005; ** *p* ≤ 0.005; * *p* ≤ 0.05 compared with control (unpaired *t*-test).

One potential mechanism by which glycolysis can be inhibited is by suppression of glucose uptake. We therefore measured uptake of the non-metabolized glucose analog 2-deoxyglucose (2DG) with or without Dpep treatment (24 h) by multiple tumor cell lines. A375, MDA-MB-231, LN229, GBM22, glioblastoma line T98G and lung carcinoma line A549 all responded to Dpep with significantly depressed glucose uptake (Figure 2A). In contrast to lines in which Dpep suppressed glycolysis, lines that did not show this response (HCT116 and MCF7) did not display a significant blockade of glucose uptake by Dpep (Figure 2B). Treatment of A375 cells with Dpep-mut failed to produce an effect, again indicating that an intact leucine zipper is required for activity and ruling out a non-specific action of the peptide (Figure 2C). Finally, non-transformed MCF10A cells, which do not undergo cell death in response to Dpep [12], showed no effect of the peptide on glucose uptake (Figure 2D). Taken together, these observations suggest that Dpep-mediated inhibition of glucose uptake and of glycolysis by tumor cells are context-dependent and closely linked.

### 3.3. Dpep Elevates TXNIP mRNA Levels in Lines with Reduced Glycolysis and Glucose Uptake, But Not in Lines without These Responses

**Figure 3:**
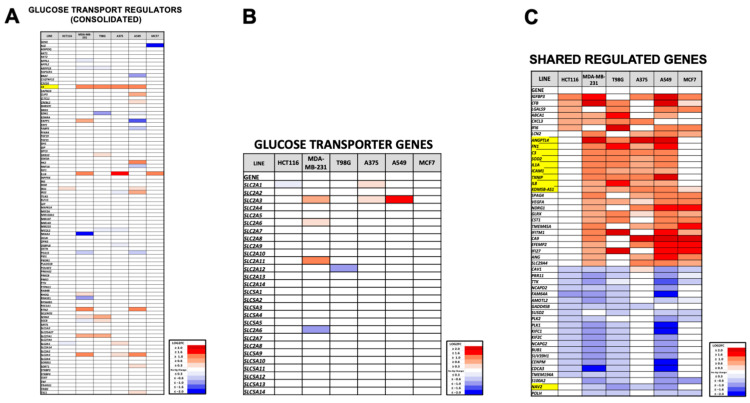
Analysis of Plate-seq data [16] identifies *TXNIP* as a potential Dpep-regulated gene that mediates cell context-dependent effects of Dpep on glucose uptake and glycolysis. (**A**) Heat map of fold-changes in expression of genes related to glucose import promoted by Dpep in HCT116, MDA-MB-231, T98G, A375, A549 and MCF7 cells. The gene list was compiled as described in the text. (**B**) Heat map as in panel A for Dpep-responsive genes encoding glucose transporters. (**C**) Heat map as in panel A of genes regulated by Dpep in at least 4 of the 6 assessed cell lines. Genes highlighted in yellow fulfill criteria of being regulated in MDA-MB-231, T98G, A375 and A549 cells, but not in HCT116 and MCF7 cells.

We next sought to uncover the potential mechanism underlying the context-dependent effects of Dpep on glycolysis and glucose uptake responses. We examined differential gene expression data previously derived by Plate-seq analysis of six different tumor cell lines (HCT116, MDA-MB-231, T98G, A375, A549 and MCF7) treated with or without 20 µM Dpep for 48 h [16]. The findings from the latter study indicated both context-dependent and shared transcriptional responses to the peptide. In particular, we searched for genes that were differentially regulated (FDR < 0.05) in the group in which Dpep suppressed glucose uptake and glycolysis (MDA-MB-231, T98G, A375, A549) vs. the group (HCT1116 and MCF7) without such a response. A list of genes related to glucose transport and its regulation was compiled from gene sets (GOMF_D_Glucose transmembrane_transporter_activity v2023.2; GOBP_Regulation_of_glucose_transmembrane_transport.v2023.2; GOBP_Regulation_of_glucose_import.v2023.2; GOBP_glucose_import_across_plasma_membrane.v2023.2; GOBP_glucose_import.v2023.2; GOBP_positive_regulation_of_glucose_transmembrane_transport.v2023.2; GOBP_negative_regulation_of_glucose_import.v2023.2; and Reactome_cellular_hexose_transport) in the GSEA database [26,27], which was then scored for Dpep responses in each cell line (Figure 3A). The only gene in this list fulfilling our criteria was *C3* (complement C3), which was elevated in the four cell lines by >2-fold. However, C3 is reported to stimulate glucose uptake via conversion to acylation-stimulating protein/C3adesArg [28]. We also compared regulation by Dpep of members of the SLC2A and SLC5A glucose transporter families across our six cell lines (Figure 3B). In this case, there was no pattern of regulation that suggested the direct involvement of such genes in our observed actions of Dpep. We additionally compiled a list of genes from the GSEA database related to glycolysis and its regulation (Kegg glycolysis gluconeogenesis; Reactome_glucose_metabolism; Kegg_medicus_reference_glycolysis; and WP_Aerobic_glycolysis (Supplementary Figure S1). Here, again, there were no genes matching our criteria. We further examined the Hallmark Glycolysis Gene Set for additional genes associated with this activity (Supplementary Figure S2). Of these genes, only *ANGPTL4* was regulated (upregulated) in lines showing Dpep-inhibited glucose uptake/glycolysis and not in lines showing such inhibition. However, the literature indicates that ANGPLT4 activates glucose uptake [29] and either stimulates [30] or has no effect [31] on glycolysis.

We hypothesized that Dpep might regulate genes relevant to glucose uptake and glycolysis that were not included in the above gene sets. We again searched for genes regulated in those lines showing Dpep-repressed glucose uptake/glycolysis, but not in those without this response. Ten genes fulfilled these criteria, including the aforementioned *C3* and *ANGPTL4* (Figure 3C). Among the remaining genes that were identified, upregulated *SOD2* and *IL8* are reported to promote rather than inhibit glucose transport/glycolysis [32,33,34], while we found no reports regarding such activities for upregulated *FN1*, *IL1A*, *ICAM1* and *KDM5B-AS1* or for downregulated *NAV2.* In contrast, for the remaining upregulated gene, the tumor suppressor *TXNIP* (thioredoxin-interacting protein), a robust literature describes inhibition of glucose uptake [35,36,37,38,39,40] and glycolysis [40,41,42,43].

### 3.4. Dpep Upregulates TXNIP Protein in Lines with Upregulated TXNIP Transcripts

**Figure 4:**
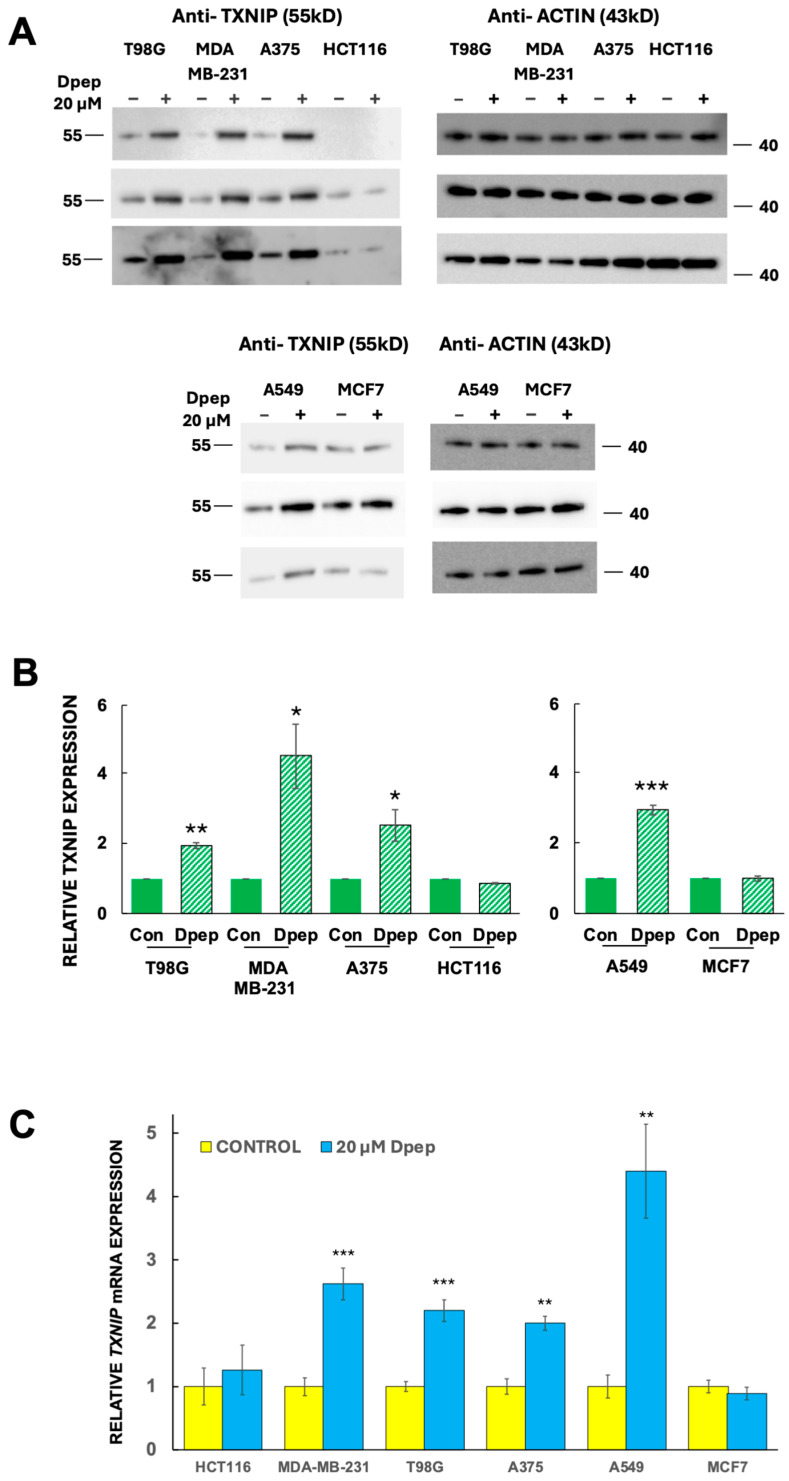
Dpep upregulates TXNIP protein in a cell context-dependent manner. (**A**) Western blot analysis of TXNIP protein expression in a panel of cancer cell lines (T98G, MDA-MB-231, A375, HCT116, A549 and MCF7) treated with or without Dpep (20 µM) for 2 days. Results from 3 independent experiments are shown. (**B**) Quantification of Western blot data for TXNIP expression as shown in panel (**A**). Data are normalized against actin expression in each experiment. Values represent means ± SEM. *** *p* ≤ 0.0005; ** *p* ≤ 0.005; * *p* ≤ 0.05 compared with control (unpaired *t*-test). (**C**) Relative *TXNIP* mRNA expression in control and Dpep-treated (20 µM, 48 h) cell lines as indicated. In each case, levels are normalized so that expression in control cells is set to 1. Values represent means ± SEM (n = 5–6). *** *p* ≤ 0.0005; ** *p* ≤ 0.005 compared with control (unpaired *t*-test). mRNA levels are from data described in Reference [16].

We next queried whether upregulation of *TXNIP* transcripts by Dpep is accompanied by elevation of TXNIP protein levels, and if so, whether this is selective. Western immunoblots of our panel of six cell lines revealed elevation of TXNIP protein by Dpep (20 µM, 48 h) in lines (MDA-MB-231, T98G, A375 and A549) characterized by *TXNIP* induction and by Dpep-promoted inhibition of glucose uptake/glycolysis (Figure 4A,B; full blots shown in Supplementary Figure S3). In contrast, there were no such effects in lines HCT116 and MCF7, in which Dpep does not regulate *TXNIP* mRNA or glucose uptake/glycolysis (Figure 4A,B). Such changes in protein expression roughly paralleled those seen for mRNA expression as quantified in Figure 4B,C. Thus, Dpep appears to induce both *TXNIP* transcripts and TXNIP protein in both shared and context-dependent manners and in lines in which it suppresses glucose uptake and glycolysis.

### 3.5. TXNIP Is Required for the Effects of Dpep on Glucose Uptake and Glycolysis and on Cell Survival

**Figure 5:**
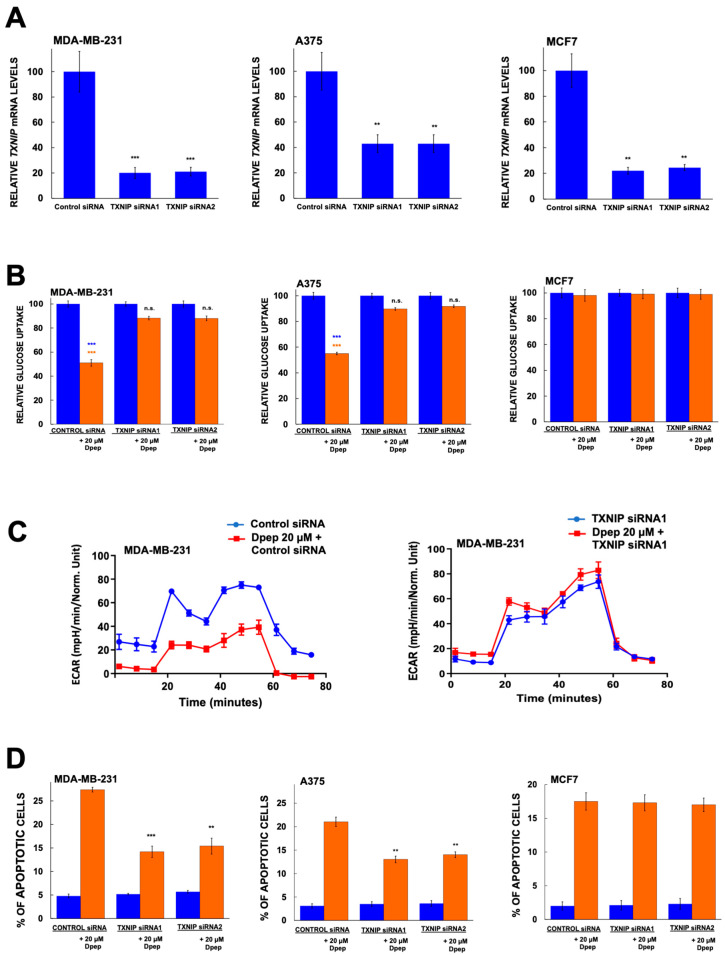
TXNIP is required for the effects of Dpep on glucose uptake, glycolysis and cell survival in a cell context-dependent manner. (**A**) Knockdown efficacy of TXNIP-targeting siRNAs in MDA-MB-231, A375 and MCF7 cells. Cells were treated with the indicated siRNAs for 2 days and then assessed for relative levels of *TXNIP* mRNA. Data are from 2–3 independent experiments, each in triplicate, and are presented as mean ± SEM. *** *p* ≤ 0.0005; ** *p* ≤ 0.005 compared with control (unpaired *t*-test). (**B**) TXNIP knockdown suppresses the effects of Dpep treatment on inhibition of glucose uptake by MDA-MB-231 and A375 cells, but not by MCF7 cells. Data are from 2–3 independent experiments, each in duplicate. *** *p* ≤ 0.0005 compared to control siRNA without Dpep treatment (unpaired *t*-test). *** *p* ≤ 0.0005 compared with TXNIP siRNA1 and siRNA2 plus treatment with 20 µM Dpep (unpaired *t*-test). n.s., not significant (*p* > 0.05). (**C**) TXNIP knockdown reverses the effect of Dpep treatment on glycolysis in MDA-MB-231 cells. Data represent mean values ± SD of three replicate cultures. (**D**) TXNIP knockdown inhibits the effect of Dpep treatment on death of MDA-MB-231 and A375 cells, but not MCF7 cells. Values are presented as mean ± SEM for 2–3 independent experiments, each in triplicate. *** *p* ≤ 0.0005; ** *p* ≤ 0.005 compared with control (unpaired *t*-test).

Our observations show a correlation between TXNIP induction by Dpep and Dpep-promoted suppression of glucose uptake. To test whether the two findings are mechanistically linked, we monitored these activities in cells in which *TXNIP* expression was down-regulated (60–80%) by two different siRNAs (Figure 5A). For these studies, we used two lines in which Dpep suppresses glucose uptake and glycolysis and induces *TXNIP* (MDA-MB-231 and A375) and one line (MCF7) in which these responses do not occur (knockdown in HCT116 cells was not successful).

TXNIP knockdown significantly blocked the effect of Dpep on glucose uptake in MDA-MD-231 and A375 cells and had no such effect on MCF7 cells (Figure 5B). We also monitored the effect of TXNIP knockdown on glycolysis in MDA-MD-231 cells and also observed reversal of Dpep-promoted suppression of this activity (Figure 5C).

TXNIP has been characterized as a tumor suppressor in multiple types of cancers [35,36,37,38,44]. This action is at least in part likely due to its suppression of glucose uptake and glycolysis. We therefore used siRNA-mediated TXNIP knockdown to assess whether the effects of Dpep on cancer cell survival are mediated by TXNIP. TXNIP knockdown in both MDA-MB-231 and A375 cells significantly protected both lines from Dpep-promoted apoptosis (Figure 5D). In contrast, TXNIP knockdown in MCF7 cells showed no significant effect on Dpep-dependent apoptosis (Figure 5D). These findings indicate that TXNIP contributes to the promotion of apoptosis in those cancer lines in which it is induced by Dpep.

### 3.6. Dpep Shows Additive to Synergistic Activity with Inhibitors of Oxidative Phosphorylation

**Figure 6:**
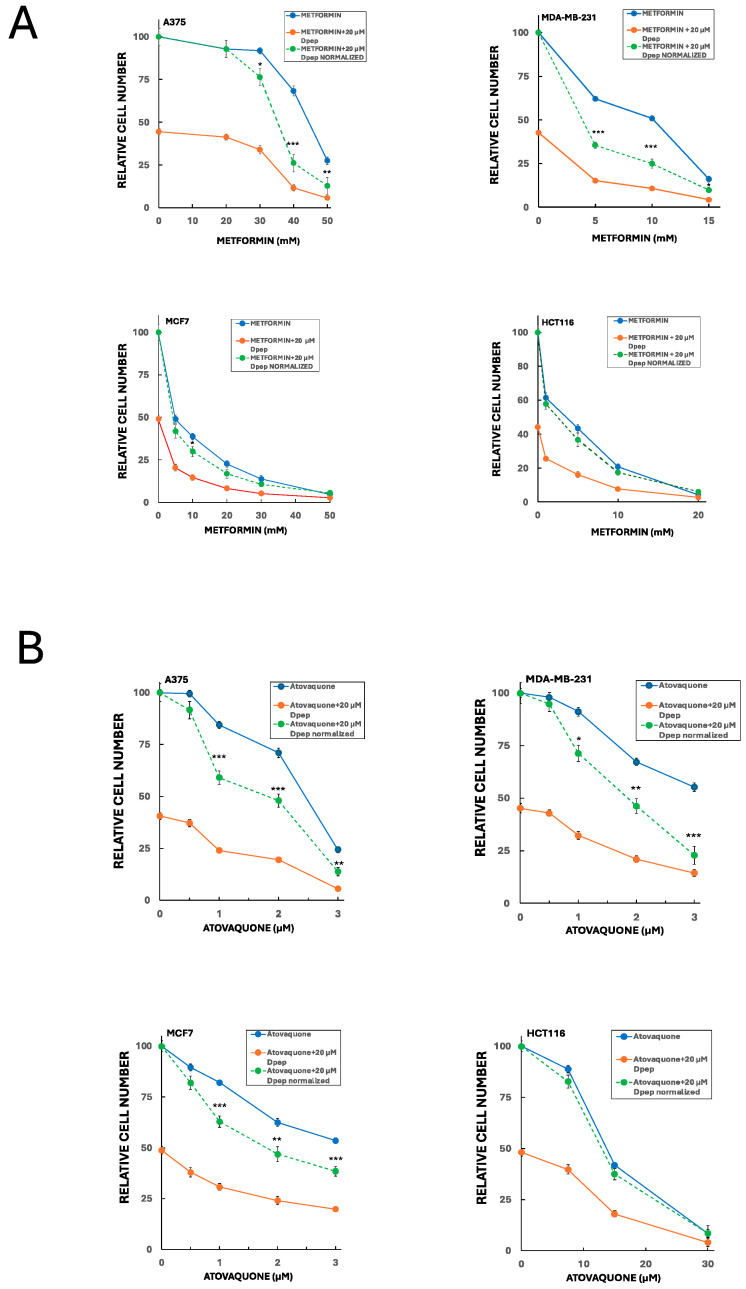
Combination of Dpep with inhibitors of oxidative phosphorylation (metformin and atovaquone) shows additive to synergistic activity in a cell context-dependent manner. (**A**) A375, MDA-MB-231, MCF7 and HCT116 cells were treated for 5 days with vehicle, 20 µM Dpep, indicated concentrations of metformin or 20 µM Dpep plus indicated concentrations of metformin and assessed for cell number. In the case of Dpep plus metformin, the data are presented both as observed values (red line) and as values normalized relative to cell numbers for Dpep alone (green dotted line). Values of the normalized data falling significantly below values for metformin alone (blue line) indicate synergy; values not significantly different for the two lines indicate additivity. Values are expressed as means ± SEM for 2–3 independent experiments, each carried out in triplicate. *** *p* ≤ 0.0005 compared to the corresponding value for metformin alone; ** *p* ≤ 0.005 compared to the corresponding value for metformin alone; * *p* ≤ 0.05 compared to the corresponding value for metformin alone. (**B**) As in (**A**), except that atovaquone was employed rather than metformin.

Our past findings revealed that Dpep performs well in combination with a range of cancer treatments in which it shows additive to synergistic actions [12]. Because Dpep suppresses glucose uptake and glycolysis, we reasoned that it might combine effectively in a complementary fashion with drugs described to interfere with oxidative phosphorylation. We therefore tested its suppression of cancer cell growth/survival in combination with metformin and atovaquone, two clinically employed drugs with favorable safety profiles that have been shown to interfere with oxidative metabolism in multiple cancers and to suppress tumor growth both in vivo and in vitro [45,46,47,48,49,50,51]. To do so, we employed fixed concentrations of Dpep (10 and 20 µM) and various concentrations of metformin or atovaquone. In combination with a range of metformin concentrations, Dpep showed synergy in A375 cells at 10 (Supplementary Figure S4) and 20 µM (Figure 6A). There was also substantial synergy between 20 µM Dpep and metformin in MDA-MB-231 cells (Figure 6A). In contrast, the combination showed largely additive efficacy in HCT116 and MCF7 cells (Figure 6A and Supplementary Figure S4). Atovaquone also exhibited significant synergy with 20 µM Dpep in MDA-MD-231 and A375 cells (Figure 6B) as well as with 10 µM Dpep in these lines (supplementary Figure S5). Again, in HCT116 cells, the two drugs appeared to act additively (Figure 6B). However, in MCF7 cells, there was a significant degree of synergy between atovaquone and Dpep at both 10 (Supplementary Figure S5) and 20 µM Dpep (Figure 6B). These findings indicate that Dpep shows additive to synergistic activity in a cell context-dependent manner when combined with drugs that suppress oxidative phosphorylation such as metformin and atovaquone.

## 4. Discussion

The object of this study has been to understand how Dpep and related cell-penetrating peptides promote selective death of a wide range of tumor cell types. This information in turn has the potential to identify the most appropriate clinical targets for Dpep as well as the most suitable partners for combination therapies. To these ends, we tested a number of diverse cancer cell lines representing both those of high frequency (e.g., lung and breast) and high mortality (GBM). We observed that many, but not all, showed significant suppression of glucose uptake and glycolysis in response to Dpep. These effects appeared to be driven by upregulation of the tumor suppressor TXNIP. TXNIP mRNA and protein are upregulated in those lines that show Dpep-dependent inhibition of glucose uptake and glycolysis, but not in lines that do not show these responses. Moreover, TXNIP knockdown suppressed the effects of Dpep on glucose uptake and glycolysis and, significantly, on tumor cell survival, but again, only in lines in which it was upregulated by the peptide.

Many tumor cell types are reliant on glycolysis for survival and proliferation under aerobic conditions and are particularly dependent on this for energy production under hypoxic conditions such as those encountered in vivo [17,18,19,20]. In considering the mechanism by which Dpep suppresses glycolysis, we found that transcriptional profiling of multiple cancer lines failed to reveal consistent regulation by Dpep of genes involved in this process, as identified in multiple gene sets.

The observation that Dpep interferes with glucose uptake in those lines susceptible to inhibition of glycolysis suggests a causal mechanistic relationship in which suppression of glucose transport leads to defective glycolysis. Here, again, transcriptional profiling and gene set analysis failed to establish a consistent mechanism by which Dpep may inhibit glucose uptake, including via regulation of glucose transporters. *TXNIP* emerged as a candidate only after the identification of genes regulated in common by Dpep in lines showing suppression of glucose uptake/glycolysis and not in lines without this response. In support of this candidacy, multiple studies have documented that TXNIP, a member of the alpha-arrestin family, can block cellular glucose uptake [35,37,38,47]. One mechanism by which this occurs is via direct interaction of TXNIP with GLUT1 (encoded by *SLC2A1*) and GLUT4 (encoded by *SLC2A4*), which in turn promotes their endocytosis, thereby lowering their capacity to import glucose [52,53,54,55,56]. Both of these transporters are reported to play important roles in cancer cell metabolism [57,58,59]. These effects occur independently from TXNIP’s association with thioredoxin [60]. Such considerations suggest a model in which Dpep upregulates TXNIP that in turn directly interacts with and reduces levels of cell surface glucose transporters, and thereby glucose transport and glycolysis.

The studies here have focused on inhibition of glucose uptake and glycolysis by Dpep and the role of TXNIP in these responses. However, it must be considered that additional tumor suppressor activities have been described for TXNIP that may also contribute to Dpep-promoted cancer cell death, including promotion of cell cycle arrest, inflammation and tumor immune responses [61,62].

Our prior work has suggested that Dpep triggers both context-dependent and shared responses in tumor cells, and that this leads to the activation of multiple pathways that ultimately converge on apoptotic death [16]. The current findings clearly place TXNIP induction by Dpep in this scheme. This response is shared by multiple cancer cell types, but not by all, thus being both shared and context-dependent. Where it is induced, TXNIP appears to contribute to cell death. However, Dpep also kills tumor cells in which TXNIP is not upregulated. This is consistent with findings that Dpep disrupts multiple pathways upstream of cell death promotion [16]. For example, in HCT116 and MCF7 cells, Dpep upregulates multiple tumor suppressors and downregulates multiple oncogenes [16]. The capacity of Dpep to impact multiple cell behaviors likely contributes to its broad efficacy and therefore its promise as a therapeutic agent for treating various cancers.

The mechanisms underlying the context-dependent nature of tumor cell responses such as that of TXNIP to Dpep have yet to be thoroughly investigated. These likely reflect both the context-dependent expression of co-regulatory transcription factors and the epigenetic landscape of each individual cancer cell. It is of interest that MDA-MB-231 and MCF7 cells, though both of ductal breast cancer origin, show differential upregulation of TXNIP by Dpep. This is consistent with the rather different overall patterns of gene regulation these lines showed to Dpep as revealed by Plate-seq [16]. Whether this reflects their distinct properties (the former line is triple-negative, whereas the latter is ER- and PR-positive) remains to be seen.

It is presently unknown whether *TXNIP* is directly regulated by Dpep targets CEBPB, CEBPD or ATF5. Our prior findings indicate that Dpep triggers a context-dependent cascade of altered transcription factor expression in cancer cells [16], thus raising the possibility of an indirect transcriptional mechanism. Multiple mechanisms and pathways have been described by which *TXNIP* expression can be regulated [35,36,37,38,39,40,41,42,43,44].

Several studies indicate that CEBPB and CEBPD promote metabolic reprogramming and affect glycolysis in various cancer cell types [20,21,22,23,24,25]. Recently, Zhang et al. [25] showed evidence that CEBPB promotes glycolysis in colon cancer cells by elevating ENO1. They reported that CEBPB knockdown diminished ENO1 expression and caused a small but significant decrease in glycolysis in HCT116 cells. In the present studies, though the effect of Dpep on glycolysis did not reach significance in HC116 cells under the conditions of our experiments, it did significantly reduce the expression of *ENO1* and other glycolysis-relevant mRNAs in this and additional cancer lines (Supplementary Figures S1 and S2). Such findings suggest that Dpep and its targets such as CEBPB may affect the glycolytic metabolism of cancer cells via context-dependent regulation of genes in addition to *TXNIP.*

Our findings raise several practical considerations for the therapeutic employment of Dpep. The capacity of Dpep to diminish glucose uptake in target cells suggests that this effect may serve as a potential biomarker for Dpep responsiveness in vivo, amenable to detection by PET scan with [18F] fluoro-2-deoxyglucose. Though not all tumor cells show this response, our present data suggest that a high proportion of tumor cells exhibit Dpep-mediated inhibition of glucose uptake/glycolysis.

The effect of Dpep on glucose uptake and glycolysis further prompted us to test the effects of its combination with several clinically approved drugs that are reported to interfere with oxidative phosphorylation, and thus with a second complementary source of cellular ATP. We observed that Dpep had synergistic actions when combined with either metformin or atovaquone in lines in which Dpep upregulated TXNIP. In contrast, Dpep showed additive effects with metformin in both lines in which TXNIP was not responsive to Dpep and additive effects with atovaquone in one of two such lines tested. These results are consistent with the hypothesis that TXNIP up-regulation sensitizes cancer cells to inhibitors of oxidative phosphorylation. The finding that Dpep synergizes with atovaquone in MCF7 cells further suggests that such an effect can also happen independently of TXNIP induction in a cell context-dependent manner. In any case, we cannot rule out the possibility that additive or synergistic interaction of Dpep with metformin and atovaquone are due to activities of these drugs unrelated to their actions on oxidative metabolism.

Irrespective of mechanisms, the additivity/synergy of Dpep with metformin and atovaquone across all cell lines tested may represent an attractive combination for therapeutic use against cancers. Metformin is undergoing clinical trials for various cancers, but with issues including dose-limiting side effects [49]. Combination with Dpep, which appears to have no evident side effects in vivo, may allow for the use of safer, reduced metformin doses to achieve efficacy. Atovaquone, which is in clinical use for *Pneumocystis jiroveci* pneumonia and malaria, is also undergoing early-phase clinical trials in combination with SOC therapeutics [45]. Here, again, our data suggest that combination with Dpep may both improve efficacy and permit use at doses with minimal side effects.

In summary, our findings point to upregulated TXNIP as a context-dependent participant in cancer cell death evoked by Dpep. Among the pro-apoptotic actions of TXNIP detected in cells treated with Dpep were inhibition of glucose uptake and glycolysis. These observations may provide a potential biomarker for Dpep actions and have led us to find that Dpep can act synergistically with FDA-approved drugs metformin and atovaquone.

## 5. Conclusions

The cell-penetrating ATF5/CEBPB/CEBPD inhibitor peptide Dpep upregulates TXNIP mRNA and protein in the majority of cancer cell lines surveyed. In the lines in which it is upregulated, TXNIP leads to inhibition of glucose uptake and of glycolysis and contributes to the promotion of cell death. Dpep acts synergistically with drugs that inhibit oxidative phosphorylation to kill cancer cells.

## Data Availability

All raw and processed Plate-seq data associated with this study are available at the Gene Expression Omnibus under accession GSE244579. Other data are available from the authors upon request.

## Supplementary Materials

The following supporting information can be downloaded at https://www.mdpi.com/article/10.3390/cells13121025/s1: Figure S1: Analysis of Plate-seq data [16] for Dpep regulation of genes associated with glycolysis (consolidated gene set) in a panel of 6 cell lines; Figure S2: Analysis of Plate-seq data [16] for Dpep regulation of genes associated with glycolysis (hallmark gene set) in a panel of 6 cell lines; Figure S3: Full blots associated with Figure 4; Figure S4: Effects of 10 µM Dpep with and without combination with various concentrations of metformin on survival of A375 and MCF7 cells; Figure S5: 10 µM Dpep shows synergistic activity in combination with atovaquone on A375, MDA-MB-231 and MCF7 cells.
