# Supplementary material for: DPEP Inhibits Cancer Cell Glucose Uptake, Glycolysis and Survival by Upregulating Tumor Suppressor TXNIP
Source: Cells. 2024 Jun 12;13(12):1025. doi: 10.3390/cells13121025 (PMC11201471; doi:10.3390/cells13121025)
Supplement: Supplementary file 1 [file cells-13-01025-s001.zip › Supplementary Figure S1.pdf]

GLYCOLYSIS PATHWAYS CONSOLIDATED

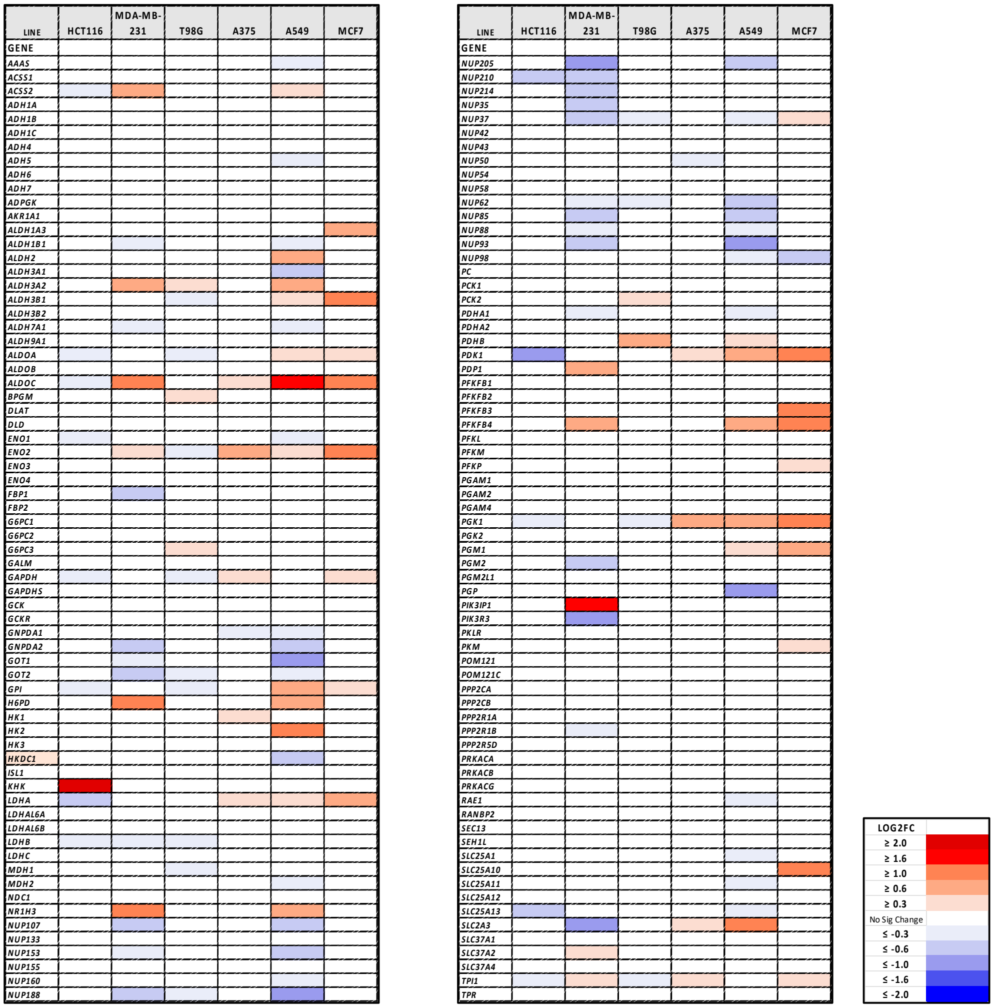

Supplementary Figure S1. Analysis of Plate-seq data [16] for Dpep regulation of genes associated with glycolysis (Consolidated gene set) in a panel of 6 cell lines. Heat map of regulated genes associated with glycolysis pathways. The list was compiled from gene sets described in the text.
