# Supplementary material for: DPEP Inhibits Cancer Cell Glucose Uptake, Glycolysis and Survival by Upregulating Tumor Suppressor TXNIP
Source: Cells. 2024 Jun 12;13(12):1025. doi: 10.3390/cells13121025 (PMC11201471; doi:10.3390/cells13121025)
Supplement: Supplementary file 1 [file cells-13-01025-s001.zip › Supplementary Figure S2.pdf]

HALLMARK PATHWAY: GLYCOLYSIS

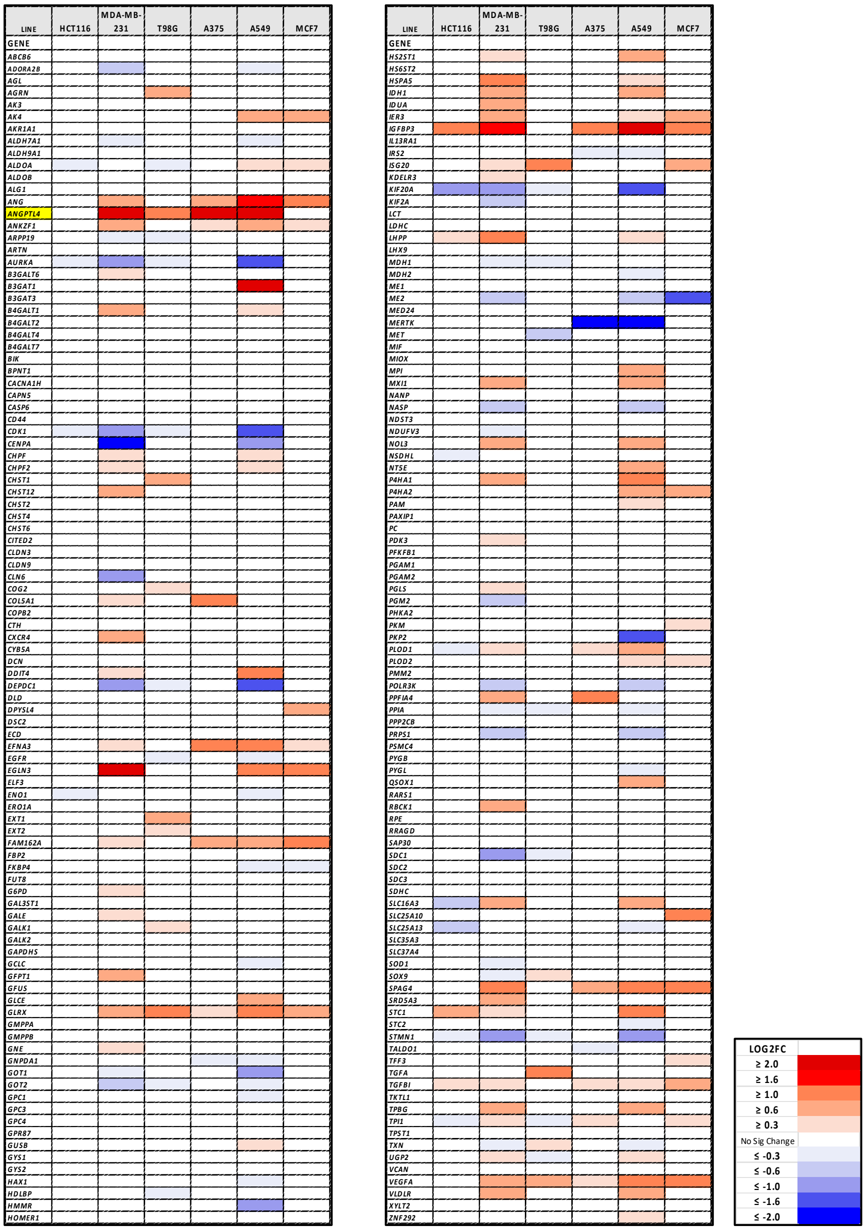

Supplementary Figure S2. Analysis of Plate-seq data [16] for Dpep regulation of genes associated with glycolysis (Hallmark gene set) in a panel of 6 cell lines. Heat map of regulated genes associated with glycolysis pathways. Regulated genes associated with glycolysis as listed in the Hallmark glycolysis gene set. Gene highlighted in yellow fulfill criteria of being regulated in MDA-MB-231, T98G, A375, and A549 cells, but not in HCT116 and MCF7 cells.
