# Supplementary material for: DPEP Inhibits Cancer Cell Glucose Uptake, Glycolysis and Survival by Upregulating Tumor Suppressor TXNIP
Source: Cells. 2024 Jun 12;13(12):1025. doi: 10.3390/cells13121025 (PMC11201471; doi:10.3390/cells13121025)
Supplement: Supplementary file 1 [file cells-13-01025-s001.zip › Supplementary Figure S4.pdf]

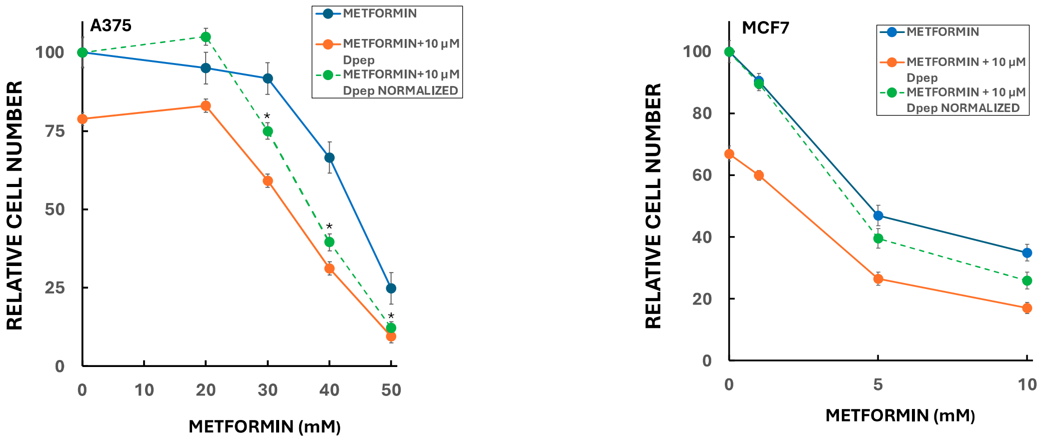

Supplementary Figure S4. Effects of 10 μM Dpep with and without combination with various concentrations of metformin on survival of A375 and MCF7 cells. Cells were treated for 5 days with vehicle, 10 μM Dpep, indicated concentrations of metformin or 10 μM Dpep plus indicated concentrations of metformin and assessed for cell number. In the case of Dpep plus metformin, the data are presented both as observed values (red line) and as values normalized relative to cell numbers for Dpep alone (green dotted line). Values of the normalized data falling significantly below values for metformin alone (blue line) indicate synergy; values not significantly different for the two lines indicate additivity. Values are expressed as means± SEM for 1-2 experiments carried out in triplicate. *p≤0.05 compared to the corresponding value for metformin alone.
