# Supplementary material for: DPEP Inhibits Cancer Cell Glucose Uptake, Glycolysis and Survival by Upregulating Tumor Suppressor TXNIP
Source: Cells. 2024 Jun 12;13(12):1025. doi: 10.3390/cells13121025 (PMC11201471; doi:10.3390/cells13121025)
Supplement: Supplementary file 1 [file cells-13-01025-s001.zip › Supplementary Figure S5.pdf]

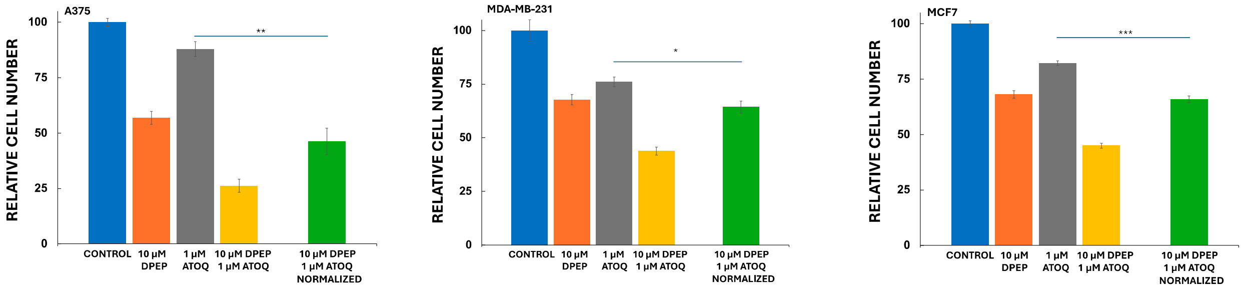

Supplementary Figure S5. 10 µM Dpep shows synergistic activity in combination with atovaquone on A375, MDA-MB-231 and MCF7 cells. A375,MDA-MB-231and MCF7 cells were treated with vehicle, 10 µM Dpep, 1 µM atovaquone, or 10 µM Dpep plus 1 µM atovaquone for 5 days and assessed for cell number. Values are expressed as means ± SEM from 1-2 independent experiments carried out in triplicate. Values represented by the green bars show data for 10 µM Dpep plus 1 µM atovaquone normalized relative to the value for Dpep alone (set to 100). Values for the normalized data below that of atovaquone alone indicate synergy. *$p \leq 0.05$ compared to the corresponding value for atovaquone alone; **$p \leq 0.005$ compared to the corresponding value for atovaquone alone; ***$p \leq 0.0005$ compared to the corresponding value for atovaquone alone.
