# Supplementary figures and images for: DPEP Inhibits Cancer Cell Glucose Uptake, Glycolysis and Survival by Upregulating Tumor Suppressor TXNIP
Source: Cells. 2024 Jun 12;13(12):1025. doi: 10.3390/cells13121025 (PMC11201471; doi:10.3390/cells13121025)

EXPT 1

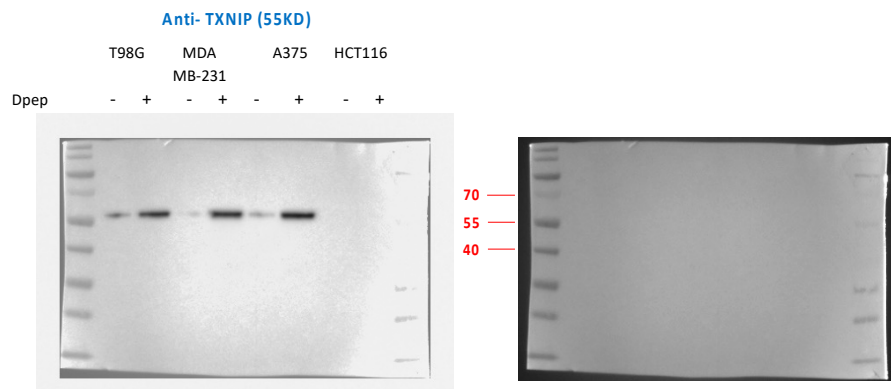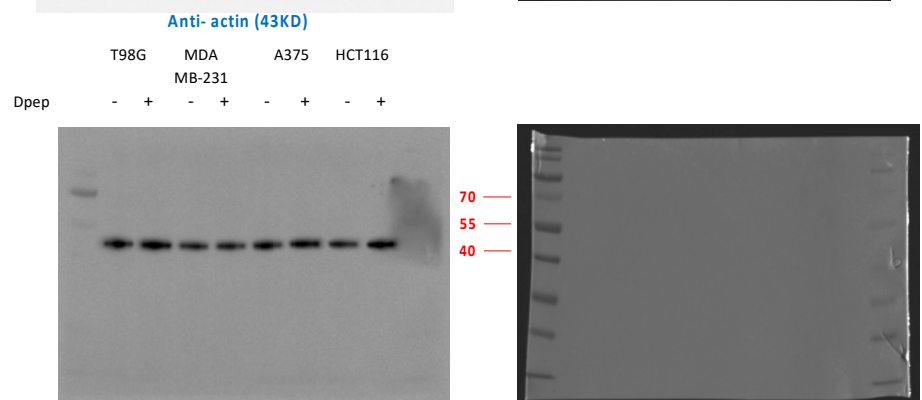

EXPT 2

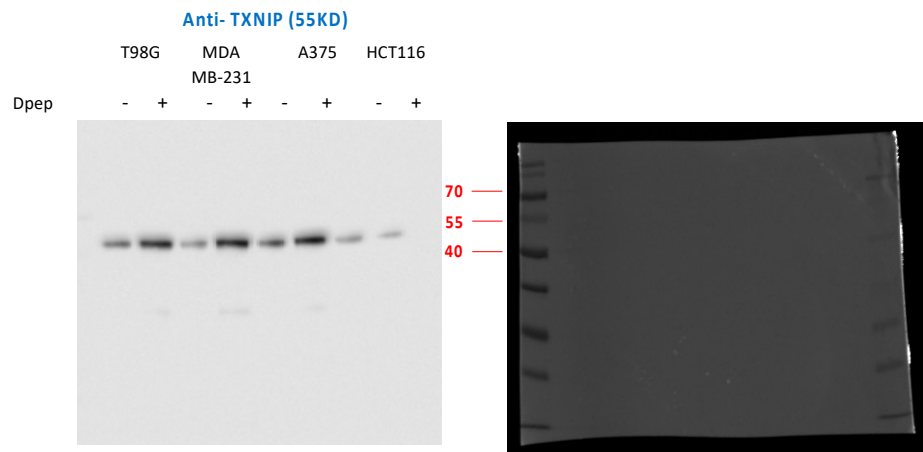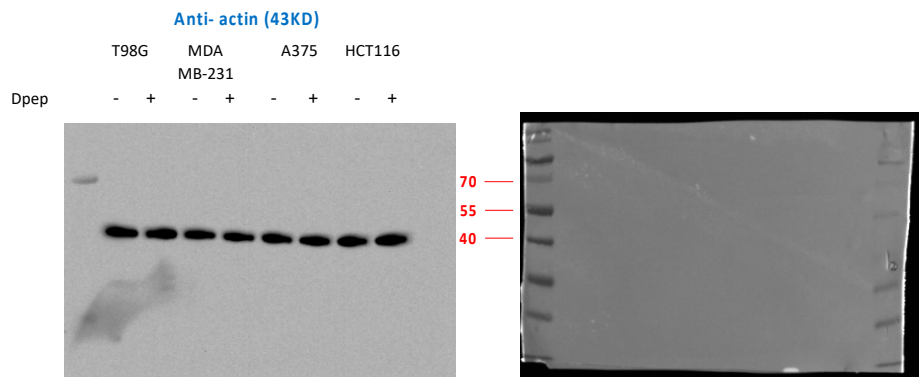

EXPT 3

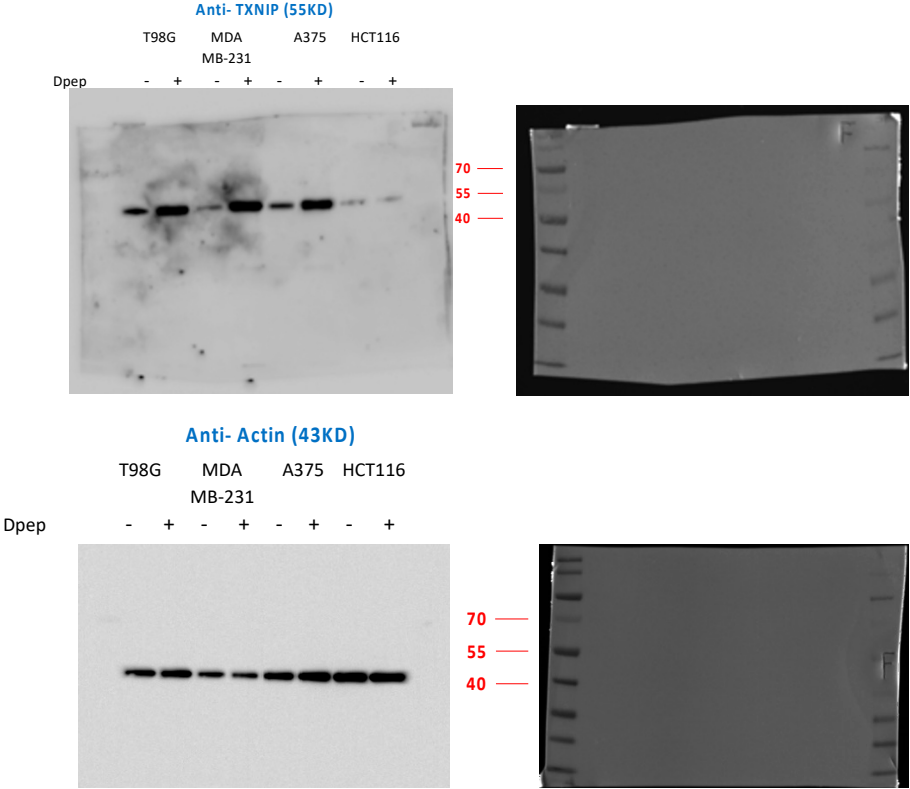

EXPTs 4,5

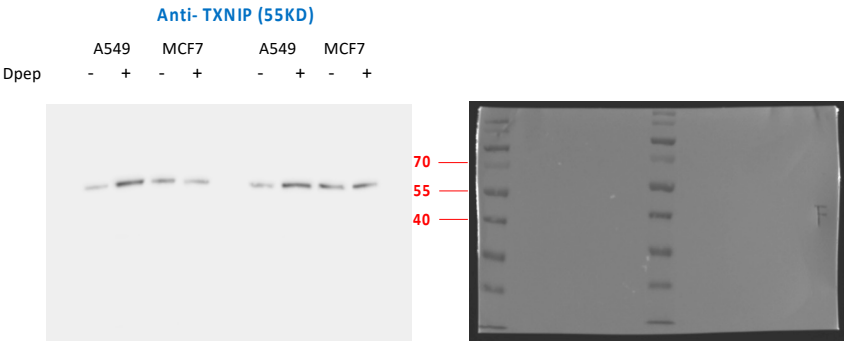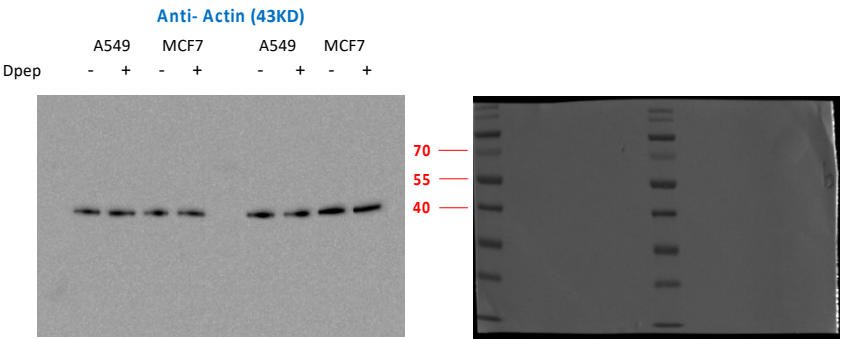

## EXPT 6

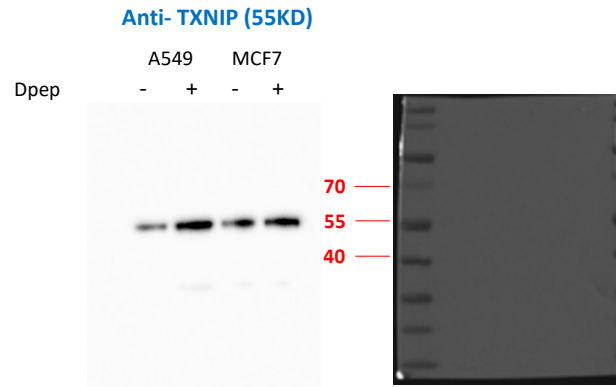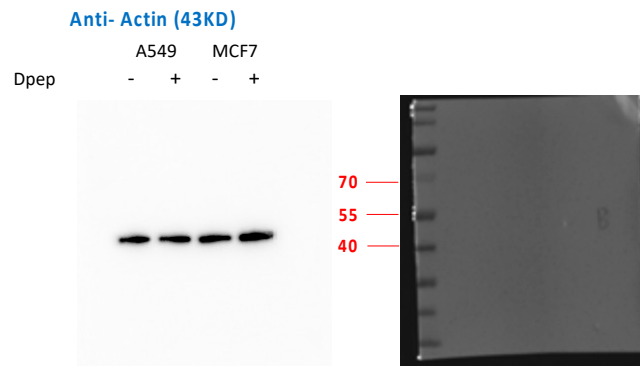

**Supplementary Figure S3.** Full blots associated with Figure 4.

Supplement: Supplementary file 1 [file cells-13-01025-s001.zip › Supplementary Figure S3.pdf]
